# Estimating Nitrogen Use Efficiency, Profitability, and Greenhouse Gas Emission Using Different Methods of Fertilization

**DOI:** 10.3389/fpls.2022.869873

**Published:** 2022-07-01

**Authors:** Muhammad Nasrullah, Lizhi Liang, Muhammad Rizwanullah, Xiuyuan Yu, Ali Majrashi, Hesham F. Alharby, Basmah M. Alharbi, Shah Fahad

**Affiliations:** ^1^College of Public Administration, Xiangtan University, Xiangtan, China; ^2^Southasia Study Center, Xiangtan University, Xiangtan, China; ^3^Department of Biology, College of Science, Taif University, Taif, Saudi Arabia; ^4^Department of Biological Sciences, Faculty of Science, King Abdulaziz University, Jeddah, Saudi Arabia; ^5^Biology Department, Faculty of Science, University of Tabuk, Tabuk, Saudi Arabia; ^6^Hainan Key Laboratory for Sustainable Utilization of Tropical Bioresource, College of Tropical Crops, Hainan University, Haikou, China; ^7^Department of Agronomy, The University of Haripur, Haripur, Pakistan

**Keywords:** nitrogen use efficiency, DP, TD, GHG emission, fertilization

## Abstract

Fertilization is a way to better use nitrogen fertilizers and increase productivity, but in another way, fertilization is also a source of anthropogenic greenhouse gas emissions. The study was carried out to measure the profitability ratio, technical efficiency, and CO_2_ from the top dressing (TD) and deep placement (DP) fertilization. The study was based on primary data, which were collected from different respondents and areas through a well-designed questionnaire. The study finds that DP fertilization is more profitable, least costly, and more efficient than TD fertilization. The finding observed that the yield of the TD growers is 727.82 kg/ha more than that of TD respondents. The efficiency score shows that to reach the 90% efficiency level, the farmers of TD need to use DP fertilization. The farmers of TD and DP can still increase their efficiency up to 12% and 9% by using the same inputs. The findings also clarify that manufacturing of synthetic nitrogen (N), direct use of N, Yield, and Area-Scaled greenhouse gas (GHG) emissions from the use of synthetic N through TD fertilization are greater than that of the DP group. The farming community needs to be aware of greenhouse gas emissions and how they can be reduced. It is also suggested that farmers need to shift toward DP fertilization to increase yield, profit, efficiency, food security, and reduce GHG emissions.

## Introduction

The rapid increase in population pressurizes the farmer community to improve productivity and overcome food shortages. With the speed at which the world population increases, it is expected that global food security and the shortage will be the foremost global challenge (Hall et al., 2017; Prosekov and Ivanova, 2018). To overcome the shortage and growing demand for food, various problems related to agricultural productivity need to be solved. Better management of land, inputs, and fertilizer application is one of the key components of improving productivity. In the race of increasing productivity and food security, farmers ignore the anthropogenic greenhouse gas (GHG) emissions from the use of fertilization and other farm activities (Shakoor et al., 2020a; Nasrullah et al., 2021a). In 2010, approximately 11% of the worldwide anthropogenic GHG emission was due to agricultural production and activity (Tubiello et al., 2013; IPCC., 2014; Shakoor et al., 2020b). Recently, many studies measure the GHG emission from different agricultural activities (Cheng et al., 2011; Maraseni and Qu, 2016; Yue et al., 2017; Maraseni et al., 2018). The application of fertilizer contributes greatly to grain production and food safety. Fertilizers play a vital role in an increase in productivity and food safety. Still, due to the influence of traditional techniques, and lack of proper knowledge and scientific guidance, many farmers use excessive fertilizers (Miao et al., 2011; Chen et al., 2016). The rapid rise in fertilizer use and nitrogen (N) use efficiency in farmland have limited the sustainable development of agriculture and caused various environmental problems (Liu et al., 2013).

For effective plant nutrition in the growing stage of the plant, deep placement of nitrogen fertilization method is healthier than the conventional fertilization (top dressing) and cultivation (Nkebiwe et al., 2016). Deep placement of nitrogen fertilizer appears to be a potential way of reducing nitrous oxide nitrogen (N_2_O–N) soil emissions (Signor et al., 2013). The N_2_O–N that is emitted from the soil during the use of urea fertilizer through deep placement was 80% lower than that emitted from the soil using top dressing (Gaihre et al., 2016). Chatterjee et al. (2018) stated that N_2_O–N emission can be reduced by using the deep placement of nitrogen because a greater proportion of fertilizer nitrogen may be kept in the soil for a longer period. Correspondingly, Chapuis-Lardy et al. (2006) argued that N_2_O–N emission can be reduced from the soil due to the N_2_O microbial consumption. Similarly, Rutkowska et al. (2017) conducted field research in Poland and stated that deep placement of nitrogen can reduce the N_2_O–N emission from the soil at a rate lower than the previous literature. Linquist et al. (2012) described that deep placement of nitrogen fertilization encourages N_2_O–N, whereas Adviento-Borbe and Linquist (2016) detected that the deep placement and broadcasting method of nitrogen fertilization produces an equal amount of N_2_O–N emission. This inconsistency in literature and results may occur due to the difference in nitrogen fertilizer use, and soil and weather conditions (Liu et al., 2020).

The agriculture sector of Pakistan plays an essential role in rural labor capital and is a major source of the national economy. The overall contribution of the agriculture sector to the gross domestic product (GDP) is 19.3%, and this sector engages 42% of the rural labor of the country (GOP (Government of Pakistan)., 2021). Maize is considered an important crop after wheat and rice, which carries a variety of important nutrients. The total share of maize is 2.4% of the total value added, which includes agribusiness at 0.5% of the GDP of Pakistan. The total production of maize was 5.70 million tons in 2017–2018, showing a decline of approximately 7% from the previous year of production (6.13 million tons). Several factors are responsible for the low yield of maize in Pakistan. Hence to improve maize productivity in Pakistan, special agro management practices and nutrition management can maximize the maize yield. Because of the high temperature and limited organic matter in Pakistan, nitrogen availability in soil for optimum plant development and growth is quite low. As a result, itis hard to imagine growing a crop without using nitrogen fertilizer. Therefore, this study was carried out with the following objectives:

- Measuring the profitability ratio of top dressing and deep placement fertilization method.
- Measuring the efficiency analysis of maize growers using top dressing and deep placement fertilization method.
- Comparing the GHG emission from the synthetic nitrogen used in top dressing and deep placement fertilization method.

## Materials and Methods

### Data and Sampling Procedure

**Figure 1:**
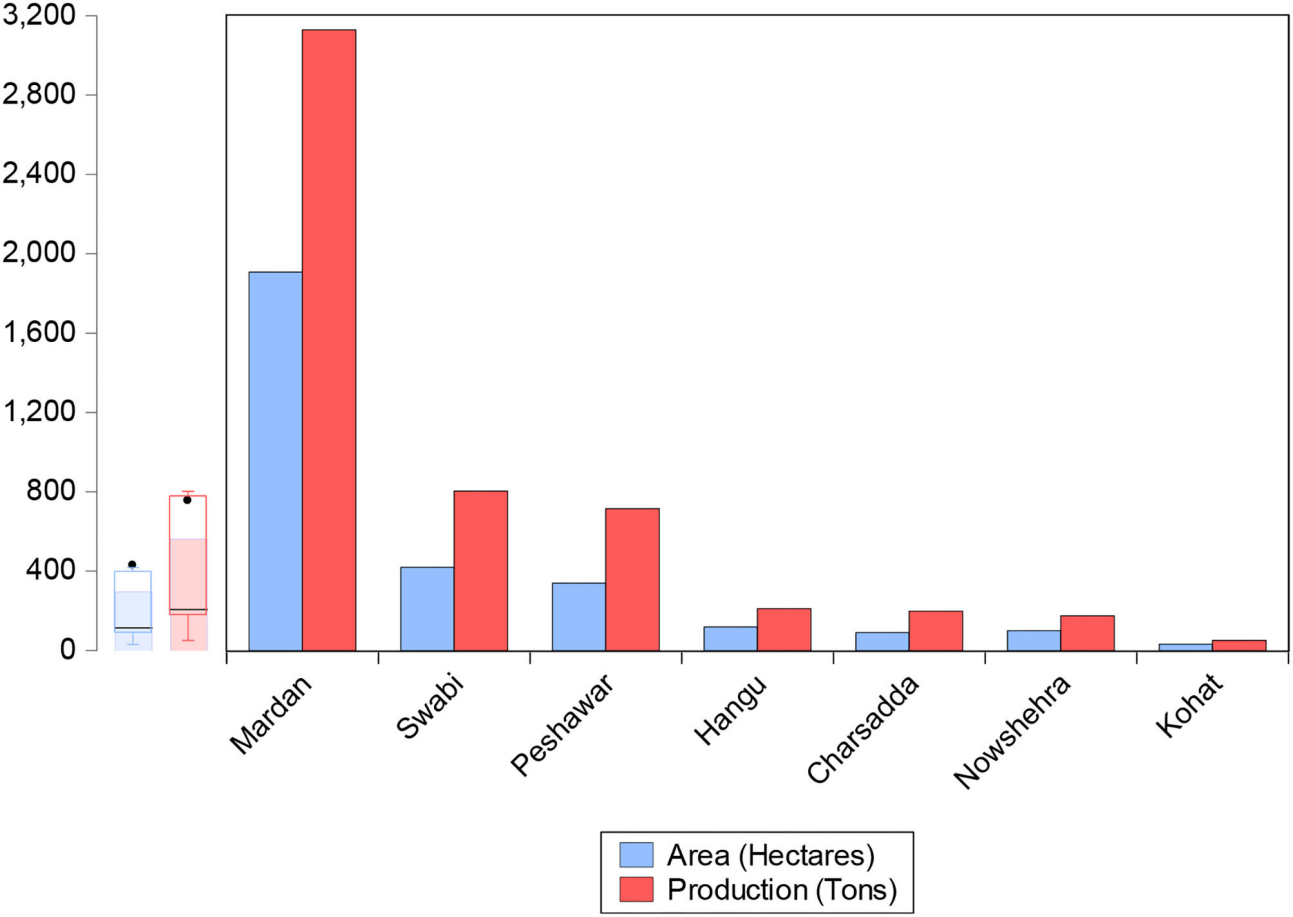
Area and production of maize in Zone C KPK (2017/2018). Khyber Pakhtunkhwa Bureau of Statistics 2017–2018 (GOKP).

**Figure 2:**
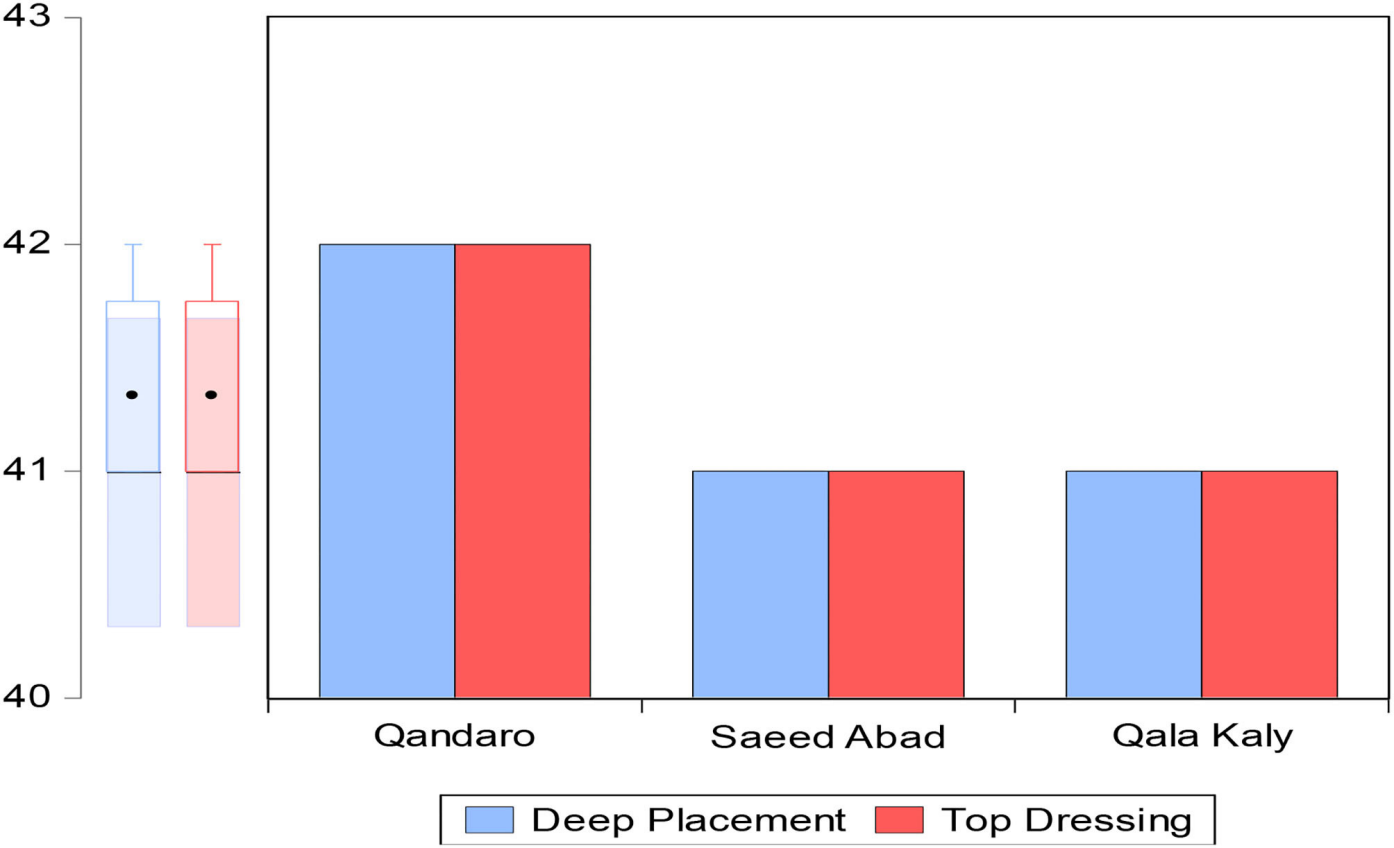
Maize growers observation in district Mardan. Survey data (2021).

Maize is an important silage crop that is grown in all provinces of Pakistan but the bulk (97%) of production comes from these two provinces, namely, Punjab and Khyber Pakhtunkhwa province (GOP (Government of Pakistan)., 2021; PARC, 2022). For the collection of maize grower data, the study used a multistage sampling technique to acquire the required sample size. In the first phase, the study was carried out in Khyber Pakhtunkhwa province based on the second highest production after Punjab. In the second phase, from the four agro-ecological zones, Mardan district was selected from Zone C among 7 districts, considering the production and number of growers as shown in Figure 1. In the third phase, Tehsil Takht Bhai was carefully chosen among the three tehsils (Mardan, Katlang, and Takht Bhai). In the fourth phase, three villages were chosen randomly, namely Qandaro, Saeed Abad, and Qala Kaly. In the last phase, a direct election approach was used to interview 125 respondents using top dressing and 125 respondents using the side-dressing fertilizer method as shown in Figure 2.

### Stochastic Frontier Production Function

Aigner et al. (1977) and Meeusen and Broeck (1977) worked independently on Farrell (1957) work and researched and developed the stochastic frontier production function. The stochastic frontier production function analysis (SFA) is unique due to the existence of a composed error term, which is different from other statistical methods. Therefore, previous studies (Sekhon et al., 2010; Musaba and Bwacha., 2014; Saddozai et al., 2015; Naveed et al., 2018; Ali et al., 2019; Jo et al., 2021) used the SFA model to measure the efficiency level due to its uniqueness. The broad method of the stochastic frontier production function can be stated as:


$$\begin{array}{l} \text{ln}(\text{yj})=\beta^{\prime }aj+vj-uj\;where\;\text{j}=1,2,\ldots \ldots \ldots ,\text{N} \end{array}$$

The yield gained from the *j*-th farmer is represented by yj, aj is a K × 1 vector having the natural log of inputs applied by the *j*-th field, the unknown parameters can be represented by β, and the symmetry error (vj) shows a random variation in the output, which is distant from the control of maize growers due to some factors. Aigner et al. (1977) stated that vj has an independent and identical distribution, denoted as *N* (0, σ^2^v), which is free from uj (random nonnegative variable). Moreover, vj is related to farm-specific features that are under the control of maize growers. These farm-specific features contribute to the technical inefficiency of maize growers, within the identical and independent distribution of the exponential form as *N* (0, σ^2^u). In addition, Coelli et al. (2005) confirmed that the stochastic frontier model takes a precise functional form, which is as follows:


$$\begin{array}{l} lnyj=\beta o+\beta 1lnxj+vj-uj\\ yj=exp\left(\beta o+\beta 1lnxj+vj-uj\right)\\ yj=exp\left(\beta o+\beta 1\;lnxj\right)\times exp\left(vj\right)\times exp\left(-uj\right)\\ exp\left(\beta o+\beta 1lnxj\right)=deterministic\;component\\ exp\left(vj\right)=error\\ exp\left(-uj\right)=inefficiency \end{array}$$

### Technical Efficiency

Coelli et al. (2005) stated that technical efficiency is the ratio between stochastic frontier output and observed output. Therefore, the technical efficiency of maize growers is as follows:


$$\begin{array}{l} TEi=\frac{qi}{\text{exp}(\beta \;xi+vi)}\\ TEi=\frac{\text{exp}(\beta \;xi+vi-ui}{\text{exp}(\beta \;xi+vi)}\\ TEi=exp(-ui) \end{array}$$

The efficiency score ranges from zero to one. A farmer is said to be more efficient, if his or her efficiency score is near one or one, and a farmer is said to be inefficient if his or her efficiency score is near zero or zero.

### Model Specification

The Cobb–Douglas stochastic frontier model was applied to enumerate the relationship between the input and output, as used in the earlier research of Battese and Coelli (1992). The model for Mardan and Swabi districts and combined districts are specified below:


$$\begin{array}{l} lnY=ln\;\beta 0+\beta 1ln\;Area+\beta 2ln\;Seed+\beta 3ln\;Tractor\\ +\beta 4ln\;Labour+\beta 5\;Irrigation+\beta 6ln\;FYM+\beta 7ln\;DAP\\ +\beta 8ln\;Urea+\beta 9ln\;Pesticides+\left(Vi--Ui\right) \end{array}$$

Where Ln is the natural logarithm; Y represents the maize yield in kilograms per hectare; βi is the parameters to be estimated; Area represents the land used for maize growing; Seed represents the seeds used in kilograms per hectare; Tractor is the tractor hours used per hectare; Labor represents the amount of labor used per hectare; Irrigation represents the number of irrigations per hectare during growth; FYM represents the Farm Yard Manure used in trolley per hectare; DAP is the Di-ammonium phosphate used in kilograms per hectare; Urea represents the urea used in kilograms per hectare; Pesticides is the amount of pesticide sprayed per hectare.

### Equation of Technical Inefficiency Estimation

The following function was used to measure the various inefficiency factors that can reduce maize production in the respondent area:


$$\begin{array}{l} Ui=\delta 0+\delta 1\;\left(Age\right)+\delta 2\;\left(Edu\right)+\delta 3\;\left(Exp\right)+\delta 4\;\left(FamS\right)\\ +\delta 5\;\left(Credit\right)+\delta 6\;\left(Exte\right)+\delta 7\;\left(Occu\right)+\delta 8\;\left(CertS\right)\\ +\delta 9\;\left(DistMarket\right)+\delta 10\;\left(OfIncome\right)+\delta 11\;\left(Tenan\right)\\ +\delta 12\;\left(Television\right)+\delta 13\;\left(Livestock\right) \end{array}$$

where Ui represents Inefficiency effect; δi represents the unknown parameters to be estimated; Age represents the age of the maize growers in years; Edu represents the education level of the maize growers in schooling years; Exp represents the experience of maize growers in years; FamS represents the family size of maize growers in numbers; Credit represents the agricultural credit obtained in rupees; Exte represents the number of visits by extension agents during the growing period; Occu is the dummy variable for respondent occupation other than farming and is equal to 1 otherwise, it is 0; CertS is a dummy variable for certified seed equal to 1; otherwise it is 0; DistMarket is the distance of the field from the local market in kilometers; Of income is a dummy variable for off-farm income and is equal to 1 otherwise, it is 0; Tenan is a dummy variable for tenancy and is equal to 1 (landowner) otherwise, it is 0 (renter); Livestock is a dummy variable for holding livestock and is equal to 1; otherwise, it is 0; Television is a dummy variable for a farmer using a Television and is equal to 1 otherwise, it is 0.

### GHG Emissions From Urea Manufacture

During the manufacturing of synthetic nitrogen, the greenhouse gases (GHG) emission includes consumption of fossil fuel, transportation, ammonia synthesis, mining, and changing ammonia to various artificial N fertilizer products (Zhang et al., 2013). For the GHG emission during the manufacturing of urea fertilizer, this study used the local GHG emission factors for urea fertilizer in Pakistan. Mir and Ijaz (2016) estimated the GHG emission factors during the manufacturing of urea fertilizers in Pakistan. The average level of GHG emission during the manufacturing of urea was estimated as 1.5 kg CO_2_ emission from the manufacturing of 1 kg of urea. Therefore, the GHG emission during the manufacturing of urea used by the top dressing and deep placement is calculated by using the following equation:


$$\begin{array}{l} M_{E}=A_{u}\times A_{rea}\times E_{Factor} \end{array}$$

Where M_E_ signifies the GHG (kg CO_2_-equal which means describing different greenhouse gases in a common unit) emission from the manufacturing of urea which is used during maize production, A_u_ signifies the amount of urea used in kilograms during maize production. A_rea_ signifies the area under the maize production and E_Factor_ is the estimated factor (1.5 kg CO_2_/kg urea) for urea in Pakistan [Mir and Ijaz (2016)]. The study also applies an ordinary least square method to measure the elasticity of variables using the following equation:


$$\begin{array}{l} Y=\beta 0+\beta_{1}X_{1}+\beta_{2}X_{2}+\varepsilon_{i} \end{array}$$

Y represents the manufacturing emissions in kg CO_2_eq, X_1_ represents the area under maize in hectares and X2 represents the amount of urea in kg, β_1_ *and* β_2_ are the slopes of X1 and X2, β_0_ in the intercept of the model and ε_*i*_ *is a* random error of the model.

### Direct N_2_O Emissions From Urea Fertilization

The direct nitrous oxide (N_2_O) emissions from the use of nitrogen were estimated from the IPCC guidelines for national GHG inventories (IPCC, 2006; tier 1) pooled with the crop-specific direct N_2_O emission and agro-region factors for the upland field were 0.010 kg N_2_O-N/kg of nitrogen (IPCC, 2006). Whereas, the amount of nitrogen available in urea is 46%. Hence, to measure the direct N_2_O-N emission from the fertilization of urea used by the respondent is 0.01(1%) kg N_2_O-N/ kg of urea. The emission factor value obtained from the IPCC, 2006 equation for the direct N_2_O-N emission from the managed soil is as follows:


$$\begin{array}{l} N_{2}O_{Direct}-N=N_{2}O-N_{N\;inputs}+N_{2}O-N_{os}+N_{2}O-N_{PRP} \end{array}$$

Where *N*_2_*O*_*Direct*_ − *N* is the direct N_2_O–N emissions, *N*_2_*O* − *N*_*N inputs*_ is the N_2_O–N emissions from N inputs, *N*_2_*O*−*N*_*os*_ is the N_2_O–N emissions from managed organic soils, and *N*_2_*O* − *N*_*PRP*_ is the N_2_O–N emissions from urine and dung inputs. The direct emission from the urea used in the study area can be measured by using the following formulation:


$$\begin{array}{l} D_{E}=A_{N}\times A_{rea}\times E_{Factor}\times 44/28 \end{array}$$

Where D_E_ signifies the N_2_O (kg N_2_O) emission from the application of urea used for maize in the respondent area, A_N_ signifies the amount of nitrogen fertilizer used by the respondent in kilograms during maize production whereas A_rea_ signifies the area under the maize production, E_Factor_ is the estimated factor of N_2_O–N (0.010 kg N_2_O–N/kg of nitrogen) and the fraction 44/28 converts N to N_2_O. The study also applies an ordinary least square method to measure the elasticity of variables using the following equation:


$$\begin{array}{l} Y=\beta 0+\beta_{1}X_{1}+\beta_{2}X_{2}+\varepsilon_{i} \end{array}$$

Y represents the manufacturing emissions in kg N_2_O–N, X_1_ represents the area under maize in hectares, and X2 represents the amount of synthetic N in Kg, β_1_ *and* β_2_ are the slopes of X1 and X2, β_0_ in the intercept of the model, and ε_*i*_is a random error of the model.

The yield-scaled and area-scaled (kg CO_2_-eq/ha) GHG emission from the use of urea for maize in the respondent area were estimated accordingly:


$$\begin{array}{l} Yield-scaled\;GHG\;emissions=\left[ME+\left(DE\times 298\right)\right]/Yield\\ Area-scaled\;GHG\;emissions=\left[ME+\left(DE\times 298\right)\right]/Area \end{array}$$

Where 298 is the radiative forcing constant of N_2_O relative to CO_2_ at a 100-year time horizon (Forster et al., 2007; IPCC, 2007; Sosulski et al., 2020).

## Results

### Descriptive Statistics of Variables

The descriptive statistics of the main variables used in the SFA model are shown in Table 1. The estimated average maize yield of top dressing and deep placement fertilization practices was 3,161.86 and 3,889.68 kg/ha. The estimate results show a significant difference in the average yield of maize in the respondent area. The paired *t*-test value proved a significant mean difference in maize yield (727 kg/ha) of deep placement and top-dressing fertilization. The average seed used by the top-dressing and deep placement group was 9.96 and 9.36 kg/ha with a significant *t*-value (1.85). The *t*-ratio at a 10% significance level shows that the seed used by the top-dressing group is 0.6 kg/ha more than the deep placement group. Likewise, the average labor used by TD and DP was 19.10 and 18.46 person/ha with an insignificant *t*-value. This result implies that both TD and DP use the same number of labor per hectare. Similarly, the mean value of land used by the respondent in the study area for top and deep placement fertilization was observed at 2.22 and 2.16 ha (1 hectare = 2.47 acres) showing that the area under maize used in both kinds of fertilization is almost the same.

**Table 1:** Descriptive statistic of major variables used in the SFA model.

	**Top Dressing**	**Deep placement**	**Total Sample**	
**Variables**	**Mean ±DP (Min–Max)**	**Mean ±DP** **(Min–Max)**	**Mean ±DP (Min–Max)**	***t*** **-test**
Yield (kg ha^−1^)	3,161.86 ± 685.48 (2,125–4,794)	3,889.68 ± 715.79 (2,210–4954)	3,525.77 ± 788.74 (2,125–4,954)	727.82^*^
Seed (kg ha^−1^)	9.96± 2.74 (6.50–15.50)	9.36 ± 2.06 (5.50–13.50)	9.66 ± 2.44 (5.50–15.50)	1.85^***^
Labor (No ha^−1^)	19.10 ± 4.03 (12.00–25.00)	18.46 ± 3.55 (12.00–25.00)	18.84 ± 3.87 (12.00–25.00)	0.50
Area (Hectare)	2.22 ± 1.00 (0.80–4.00)	2.16 ± 0.97 (0.60 - 3.80)	2.19 ± 0.99 (0.60–4.00)	0.06
Irrigation (Frequency)	5.58 ± 0.64 (5.00–7.00)	5.99 ± 0.70 (5.00–7.00)	5.79 ± 0.70 (5.00–7.00)	0.41^*^
FYM (Trolley)	1.95 ± 1.34 (0.00–4.00)	1.93 ± 1.32 (0.00–4.00)	1.951 ± 1.25 (0.00–4.00)	0.02
DAP (kg ha^−1^)	37.86 ± 8.36 (25.00–50.00)	39.32 ± 7.04 (25.00–50.00)	38.59 ± 7.70 (25.00–50.00)	−1.47
Urea (kg ha^−1^)	150.24 ± 58.74 (62.00–249.00)	108.82 ± 39.57 (47.00–200.00)	129.53 ± 54.12 (47.00–249.00)	41.14^*^
Tractor (hrs ha^−1^)	9.75 ± 1.79 (7.00–12.50)	10.03 ± 1.99 (7.00–14.00)	9.87 + 1.89 (7.00–14.00)	0.33
Pesticide (No ha^−1^)	1.42 ± 0.49 (1.00–2.00)	1.42 + 0.49 (1.00–2.00)	1.42 + 0.49 (1.00–2.00)	0.01
Nitrogen use (kg ha^−1^)	69.11 ± 27.02 (28.52–114.54)	50.06 ± 18.20 (21.62**–**92.00)	59.58 ± 24.89 (21.62–114.54)	19.05^*^

*Survey data 2021. The*

*, ** and *** *shows the significant difference at 1%, 5% and 10%*.

The observed statistics show that the average irrigation applied by the TD and DP was 5.58 and 5.99 times with a significant mean difference of 0.40 times. The results show that DP group farmers use more water as compared to the TD group. The mean farmyard manure used by the TD and DP was 1.95 and 1.93 trolley (1 trolley = 500 kg of manure) ranging from 1 to 4 trolleys. It was observed that both groups of fertilization are using the same amount of FYM. The estimated descriptive mean DAP used by the TD and DP groups was 37.86 and 39.32 kg/ha, respectively. There is no significant difference in the use of DAP by both the, groups. The estimated average urea used by TD and AD respondents was 150.24 and 108.82 kg/ha respectively, with a significant mean difference of 41.41 kg/ha. The results show that the urea used in broadcasting (TD) is more as compared to the deep placement fertilization. The average tractor used by the TD and DP was 9.75 and 10.03 h/ha, respectively, while the average pesticide sprayed by the TD and DP was 1.42 times. The results show that there is no difference in the pesticides and tractors used by both the groups. The average nitrogen estimation obtained from urea for TD and DP is 69.11 and 50.06 kg/ha, respectively. The test statistics show a significant difference of 19.05 kg of nitrogen used during TD and DP fertilization.

The descriptive statistics of farmers' attributes and socio-economic factors are described in Table 2. The mean age observed for the TD and DP respondents were 44.13 and 42.54 years, respectively, while the education level was 8.27 and 7.88 schooling years, respectively. The results show that farmers' mean age and education of TD and DP groups are the same. It is also observed that the average experience of the TD and DP respondents was 13.14 and 16.29 years, whereas the average family size was 8.62 and 9.27 persons, respectively. The average amount of agriculture credit received by the TD and DP respondents observed was 80.68 × 10^3^ and 80.83 × 10^3^ Pakistani rupees, respectively, while the mean extension visit was 0.88 and 1.12 times, respectively, in the study area. Similarly, the respondents engaged in other occupation along with agriculture was observed at 0.51 and 0.39 in the TD and DP group while the mean certified seed used was 0.61 and 0.64, respectively. The mean distance between field and market was 3.15 and 3.64 km was observed for the TD and DP groups while the farmers who received their incomes from other sources were 0.49 and 0.42, respectively. The descriptive also shows that the average tenancy, television used by the respondents, and livestock were 0.50, 0.49, and 0.68 for the TD group, while 0.58, 0.37, and 0.72 were observed for the DP group.

**Table 2:** Descriptive statistics of inefficiency variable used in SFA.

	**Top Dressing**	**Deep placement**	**Total Sample**
**Variables**	**Mean ±DP (Min–Max)**	**Mean ±DP** **(Min–Max)**	**Mean ±DP (Min–Max)**
Age (Years)	44.13 ± 6.17 (28–58)	42.54 ± 6.26 (27–56)	43.33 ± 6.25 (27–58)
Education (Years)	8.27 ± 3.93 (00–14)	7.88 ± 4.29 (00–14)	8.08 ± 4.11 (00–14)
Experience (Years)	13.14 ± 7.79 (03–31)	16.29 ± 6.55 (03–26)	14.72 ± 7.35 (03–31)
Family size (Number)	8.62 ± 2.52 (05–14)	9.27 ± 2.72 (05–13)	8.95 ± 2.64 (05–14)
Credit in PKR (×10^3^)	80.68± 122.34 (00–559.39)	80.83 ± 132.24 (00–948.80)	80.76 ± 127.13 (00–948.80)
Extension agent visit (Number)	0.88 ± 0.69 (00–02)	1.12 ± 0.75 (00–02)	1.00 ± 0.73 (00–02)
Other occupation	0.51 ± 0.50 (00–01)	0.39 ± 0.49 (00–01)	0.45 ± 0.50 (00–01)
Certified seed	0.61 ± 0.49 (00–01)	0.64 ± 0.48 (00–01)	0.62 ± 0.49 (00–01)
Distance from Market (km)	3.15 ± 1.90 (0.20–6.2)	3.64 ± 1.99 (0.30–08)	3.39 ± 1.96 (0.2–08)
Off-farm income	0.49 ± 0.50 (00–01)	0.42 ± 0.50 (00–01)	0.47 ± 0.50 (00–01)
Tenancy	0.50 ± 0.50 (00–01)	0.58 ± 0.50 (00–01)	0.54 ± 0.50 (00–01)
Television	0.49 ± 0.50 (00–01)	0.37 ± 0.48 (00–01)	0.42 ± 0.49 (00–01)
Livestock	0.68 ± 0.47 (00–01)	0.72 ± 0.45 (00–01)	0.70 ± 0.46 (00–01)

*Survey data 2021*.

### Cost and Net-Return of Maize Production (Per Hectare)

The study also measures the cost and net return of top dressing and deep placement which is shown in Table 3. The estimated results show that the tractor used for plowing and seedbed preparation incurred the highest average cost of 20.14% and 20.80% of the total cost on top and deep placement while the average rental cost of the land is the second most costly input used by both groups. The cost spent on land was 19.18% of the maize total cost for the TD group while 19.57% of the total cost for the DP group. It is also observed that the mean labor cost was 14.09 and 14.18% of the total cost incurred by TD and DP respondents, respectively. The fourth highest cost input was urea in the study area. The cost incurred by TD and DP was 12.41 and 10.92% of the total cost while transportation cost was the least cost incurred by the TD and DP group. The total cost of maize production per hectare was observed at 67,787 PKR/ha on top-dressing fertilization while on side-dressing the total cost was 66,421.4 PKR/ha. The estimated results show that the net revenue gained by top dressing was 71,187.8 PKR/ha while net revenue gained by side-dressing farmers was 172,087.2 PKR/ha. The profitability ratio portrays that the deep placement fertilization method was a more profitable and least cost method as compared to the top-dressing fertilization.

**Table 3:** Average cost and net return of maize (per hectare).

			**Top dressing**			**Deep placement**		
**Variables**	**Units**	**Cost/unit(Rs)**	**Quantity**	**Cost**	**%**	**Quantity**	**Cost**	**%**
Area	hectare	13,000	1	13,000	19.18	1	13,000	19.57
Seed	Kg	600	9.96	5,976	8.82	9.36	5,616	8.46
Tractor	Hours	1,400	9.75	13,650	20.14	9.87	13,818	20.80
Labor	No:	500	19.10	9,550	14.09	18.84	9,420	14.18
Irrigation	Fixed	2,400		2,400	3.54		2,400	3.61
FYM	Trolley	2,000	1.95	3,900	5.75	1.95	3,900	5.87
DAP	Kg	160	37.86	6,057.6	8.94	38.59	6,174.4	9.30
Urea	Kg	56	150.24	8,413.4	12.41	129.53	7,253	10.92
Pesticides	Bottle	2,000	1.42	2,840	4.19	1.42	2,840	4.28
Production cost			65,787	97.05		64,421.4	96.99	
Transportation cost			2,000	2.95		2,000	3.01	
**Total cost**				**67,787**	**100**		**66,421.4**	**100**
		**Price/kg**	**Quantity**	**Revenue**		**Quantity**	**Revenue**	
**Yield**	**Kg**	**40**	**3,161.86**	**12,6474.4**		**3,889.68**	**15,5587.2**	
**By-product**				**12,500**			**16,500**	
**Total Revenue**				**138,974.4**			**172,087.2**	
**Net-Revenue**	**Total revenue - total cost**			**71,187.4**			**105,665.8**	
**Profitability ratio**	**Net revenue /total cost**			**1.05**			**1.59**	

*Survey data 2021. The bold vale shows the total estimation values of cost and net return*.

### Correlation Analysis

The correlation analysis was carried out among the variables of TD, DP, and the total sample model was carried out as shown in Tables 4–6. The estimated results show a weak correlation among all the variables input used in the study. These results verify that the impact of each variable input on the production of maize was not affected by the use of other variables. Therefore, the estimated results of the SFA model for TD, DP, and total sample model are unbiased.

**Table 4:** Pearson's correlation analysis of top-dressing fertilization.

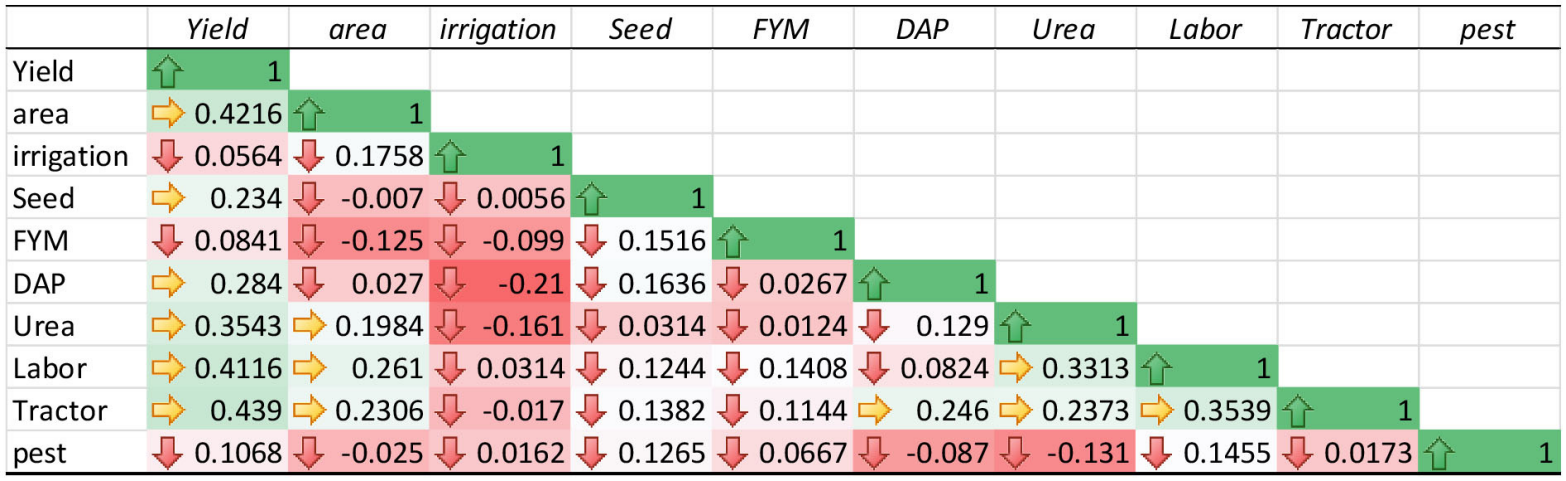

*Survey data 2021. The color green and yellow indicates the maximum and minimum value of correlation, similalrly, the green, yellow and red arrow indicates the maximum, medium and minimum correlation*.

**Table 5:** Pearson's correlation analysis of deep placement fertilization.

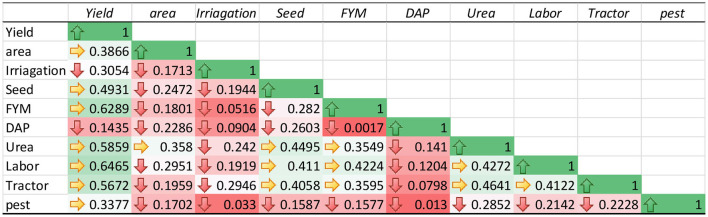

*Survey data 2021. The color green and yellow indicates the maximum and minimum value of correlation, similalrly, the green, yellow and red arrow indicates the maximum, medium and minimum correlation*.

**Table 6:** Pearson's correlation analysis of total sample model.

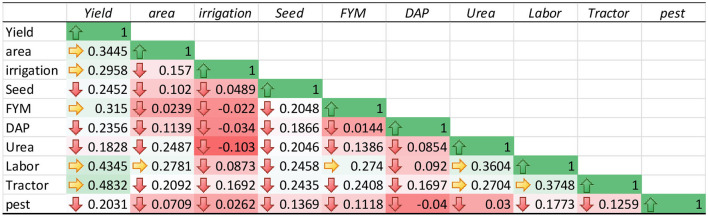

*Survey data 2021. The color green and yellow indicates the maximum and minimum value of correlation, similalrly, the green, yellow and red arrow indicates the maximum, medium and minimum correlation*.

### Estimated Results of the SFA Model for TD, DP, and Total Data

The estimated output of the stochastic frontier model for top dressing, deep placement, and total sample are shown in Table 7. The projected result of seed used by the TD, DP, and total sample respondents are statistically significant at 1, 10, and 1% significant levels, respectively. The results portray that an increase of 1% in seed rate can significantly increase the yield of TD, DP, and total sample respondents by 0.11%, 0.05%, and 0.08%. The estimated results of seed rate show that both TD and DP groups are using quality seed of maize but the elasticity of seed for TD is more than DP, which implies that the seed quality and seedbed of TD is better than DP (Saddozai et al., 2015; Nasrullah et al., 2021b). Similarly, the number of labor used for TD and DP also shows a significant effect on the maize yield at a 1% and 5% significant level. The significant impact of labor shows the better management of labor used by both groups. The results are similar to the previous studies of Ali et al. (2019) and Jo et al. (2021).

**Table 7:** Maximum likelihood estimation.

	**Top Dressing**	**Deep placement**	**Total Sample**
**Variables**	**Coefficient (Std. Error)**	**Coefficient** **(Std. Error)**	**Coefficient (Std. Error)**
Constant	5.326a (0.281)	7.054a (0.145)	6.455a (0.153)
Seed (kg ha-1)	0.112a (0.034)	0.048c (0.025)	0.081a (0.023)
Labor (No ha-1)	0.231a (0.052)	0.076b (0.034)	0.184a (0.032)
Area(Hectare)	0.064a (0.019)	0.018b (0.009)	0.035a (0.011)
Irrigation (Frequency)	0.284a (0.099)	0.113a (0.036)	0.171a (0.047)
FYM (Trolley)	0.007a (0.003)	0.005b (0.003)	0.008a (0.002)
DAP (kg ha-1)	0.096b (0.043)	0.053b (0.026)	0.071a (0.028)
Urea (kg ha-1)	0.118a (0.052)	0.064a (0.017)	0.063a (0.016)
Tractor (h ha-1)	0.162a (0.061)	0.104a (0.026)	0.097a (0.031)
Pesticide (No ha-1)	0.123a (0.029)	0.044a (0.014)	0.054a (0.016)
Top dressing			-0.115a (0.017)
Lambda (λ = σ u/σ v)	2.018	2.081	2.156
Γ= λ^2^ /1+ λ^2^	0.802	0.812	0.823
Log-likelihood	55.596	124.522	146.480

*Survey data (2021). The a, b indicates the significance level at 1% and 5% respectively*.

The area used by the respondents also plays a significant role in the production. The projected elasticity of the area shows a 0.64% and 0.02% increase in maize production of TD and DP groups if there is a 1% increase in the area. The results are coinciding with the previous studies of Nasrullah et al. (2019) and Zulfiqar et al. (2020a), which stated that soil fertility and better management of land can increase production. Likewise, the irrigation application in the projected area is also significant at a 1% significance level for both TD and DP. The results are similar to the previous studies by Traore et al. (2000) and Payero et al. (2009), which specified that on-time irrigation can increase productivity while water stress can decrease the maize production and kernel size. The Farmyard manure also shows a substantial rise in the production of maize. The maize production can be increased by 0.007% and 0.005% with an increase of 1% in FYM. Naveed et al. (2018) stated that FYM provides micronutrients to the maize crop and increases productivity.

The DAP used by the respondents has significantly improved the maize yield by 0.096% and 0.053% at a 5% significance level. Chen et al. (2020) demonstrated that DAP used for maize increases productivity. Similarly, the impact of urea is also statistically significant at a 1% significance level. The results show that a 1% increase in urea increases the maize production by 0.118% and 0.064% of TD and DP respondents. This result indicates that an accurate amount of fertilizer was used at a suitable subsidized price (Ali et al., 2019). The tractor used for seedbed preparation has an effective impact on the production of maize in the study area. The increase of 1% in tractor hours can increase the productivity by 0.162% and 0.104% of TD and DP respondents. The machinery used for inverting and plowing land had a direct relation with production (Sekhon et al., 2010; Nasrullah et al., 2019). Likewise, pesticides used by respondents in the study area significantly improve maize production by 0.123% and 0.044% with a 1% increase in pesticide use. The results are similar to the previous study by Popp et al. (2013), which stated that a reduction in crops occurs due to pests.

The dummy variable used for top dressing in the whole model is significant with a negative coefficient implying that the deep placement method is better for productivity as compared to top dressing. The variance parameter Lambda (λ) is >1 showing that our model is a good fit while Gamma (Γ) shows that the unexplained inefficiency variable can cause 80% and 81% variation in maize production of TD and DP.

### Technical Efficiency Ranges for the TD and DP Respondents

**Figure 3:**
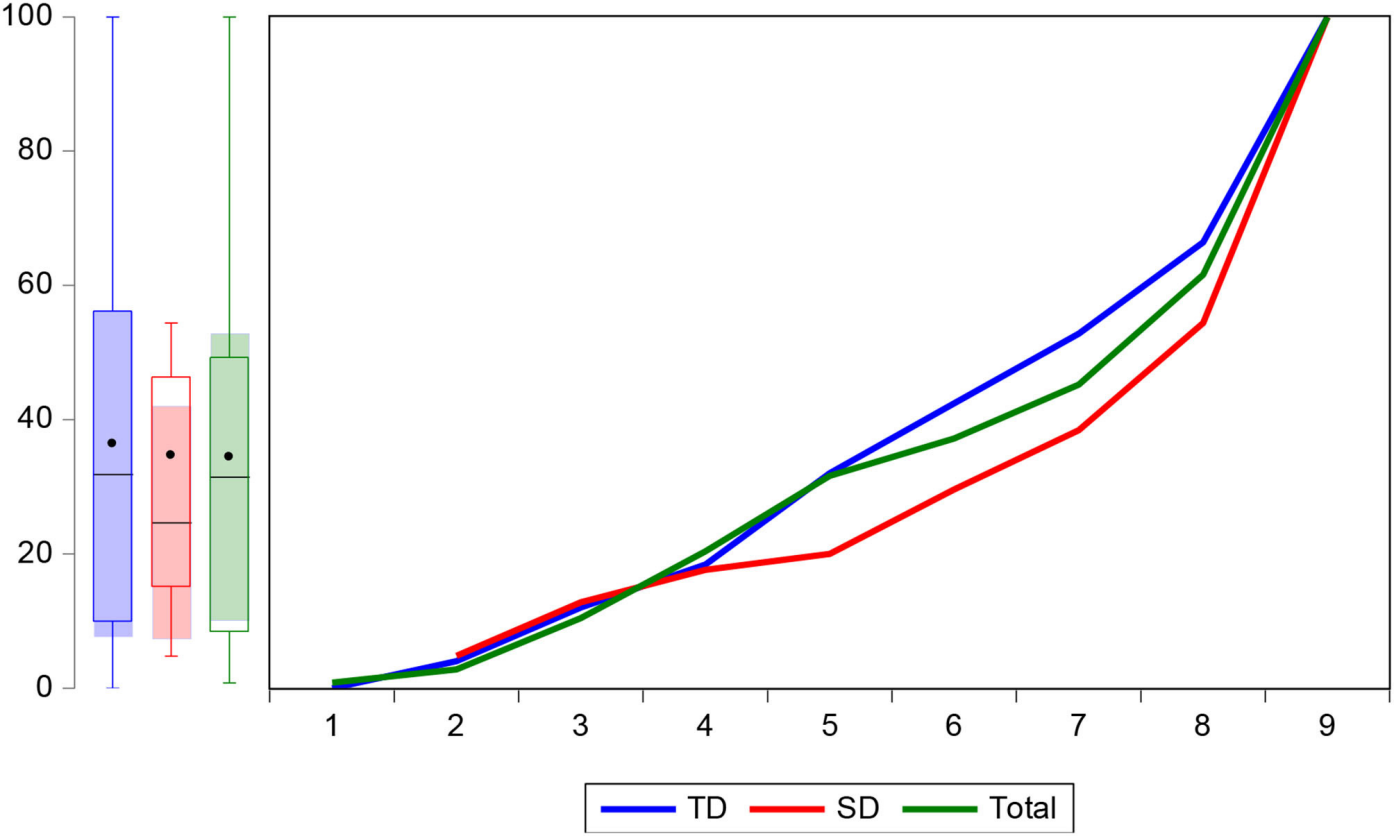
Cumulative distribution of the efficiency scores for TD and DP groups.

The ranges and frequency distribution of respondents' efficiency levels are publicized in Table 8 for the TD and DP groups. The estimated result illustrates that the mean efficiency level of TD and DP was 87 and 90%, respectively. The demonstrated results verify a substantial difference between the TD and DP efficiency levels. The projected efficiency of the DP group was 3% less than that of the TD group, representing a significant gap between the two groups. Purucker and Steinke (2020) stated that deep placement fertilization reduces nitrogen losses and increases the efficiency level. The study further shows a gap of 12% and 9% from the mean to maximum efficiency of TD and DP groups, which implies that TD and DP group can increase their efficiency up to 12 and 9%, respectively, with a given amount of inputs. Figure 3 shows that the cumulative frequency distribution of the efficiency score of the TD and DP groups was concave to the origin, which implies that some factors affect the efficiency level of farmers.

**Table 8:** Efficiency ranges of the TD and DP groups.

	**Top Dressing**		**Deep placement**		**Total Sample**	
**Efficiency Ranges**	**Frequency**	**Percentage**	**Frequency**	**Percentage**	**Frequency**	**Percentage**
55–60	0	0	0	0	2	0.8
61–65	4	4	6	4.8	5	2
66–70	8	8	10	8	19	7.6
71–75	6.4	6.4	6	4.8	25	10
76–80	13.6	13.6	3	2.4	28	11.2
81–85	10.4	10.4	12	9.6	14	5.6
86–90	10.4	10.4	11	8.8	20	8
91–95	13.6	13.6	20	16	41	16.4
96–100	33.6	34.4	57	45.6	96	38.4
Total	125	100	125	100	250	100
Mean	0.87		0.90		0.87	
Std. Dev.	0.11		0.11		0.11	
Min	0.61		0.62		0.59	
Max	0.99		0.99		0.99	

*Survey data (2021)*.

### Technical Inefficiency Estimation Model of the TD and DP Groups

The demographic, institutional, and socioeconomic factors that increase the inefficiency level of maize growers are explained in Table 9. The negative coefficient of the variable in the inefficiency model showed a positive impact on the efficiency level of farmers, while the positive sign of the coefficient showed a negative impact. The inefficiency factor age has a significant positive effect on the efficiency level of maize growers in the TD or DP groups. The increase in age showed the weak physical condition of the growers (Saddozai et al., 2015; Bayisenge et al., 2019). The education level of maize growers showed a significant effect on the efficiency level of the maize growers in the TD and DP groups at the 10% and 5% significance levels. The significant impact of education showed the improved skills of the farmers, which is a key indicator of achieving improved technology. Previous studies by Dhungana et al. (2004) and Mariano et al. (2010) stated that education plays an important role in adopting new technology while Rios and Shively (2005) stated that higher education decreases the efficiency level.

**Table 9:** Inefficiency factors of TD and DP groups.

	**Top Dressing**	**Deep placement**	**Total Sample**
**Variables**	**Coefficient (Std. Error)**	**Coefficient** **(Std. Error)**	**Coefficient (Std. Error)**
Constant	−8.125a (2.828)	−3.123c (1.921)	−5.067a (1.486)
Age (Years)	0.076b (0.037)	0.054c (0.029)	0.063a (0.023)
Education (Years)	−0.105c (0.056)	−0.084b (0.042)	−0.061c (0.035)
Experience (Years)	-0.067c (0.037)	−0.068b (0.031)	-0.062a (0.022)
Family size (Number)	−0.186c (0.102)	−0.203b (0.093)	−0.207a (0.069)
Credit in PKR (×10^3^)	0.012 (0.034)	0.031 (0.032)	0.012 (0.023)
Extension agent visit (Number)	−1.017a (0.397)	−0.583c (0.322)	−0.645a (0.232)
Other occupation	1.122b (0.510)	0.764b (0.367)	1.013a (0.325)
Certified seed	−0.958a (0.407)	−0.664c (0.388)	−0.894a (0.274)
Distance from Market (km)	0.730a (0.302)	0.251b (0.119)	0.382a (0.114)
Off-farm income	0.897b (0.447)	−0.804c (0.433)	−0.0002 (0.286)
Tenancy	−0.779b (0.396)	−0.767b (0.403)	−0.511b (0.265)
Television	−0.683c (0.396)	−0.958c (0.512)	−0.119 (0.269)
Livestock	−0.442 (0.415)	−0.080 (0.381)	−0.419 (0.274)

*Survey data, (2021). The a, b and c indicates the significance level at 1% and 5% respectively*.

The significant coefficient of farmers' experience at 10 and 5% levels for the TD and DP groups showed that farmers have a long connection with farming activities and can easily adopt advanced technology (Bayisenge et al., 2020; Zulfiqar et al., 2020b). Similarly, the family size of the respondents is also significant for TD and DP. The noteworthy impact of family size is similar to that in the study by Coelli et al. (2002), which stated that a large family is labor intensive. Institutional factor extension agent visits to maize growers had a significant impact on maize production at the 1 and 10% level for the TD and DP groups. The results are similar to those of a previous study by Alene and Hassan (2006), which stated that a weak connection of extension agents with farmers decreased the production level of the growers. The estimated coefficient of main occupation other than agriculture had an inverse impact on the production level of the maize farmers for both groups at the 5% level. Chen (2017) stated that farmers engaged in other occupations (part-time farming) along with farming reduces the productivity and efficiency level of growers.

The estimated elasticity of certified seeds for the TD and DP groups were 0.96 and 0.66, respectively, which were significant at the 1 and 10% levels. The estimated results of the certified seeds were in line with the results of the study by Musaba and Bwacha (2014). Distance of field from the market significantly decreases the efficiency level of growers. The results were similar to those of the previous study by Ali et al. (2019) and Nasrullah et al. (2020). The increase in off-farm income significantly decreases the efficiency level of both groups. Off-farm income activities reduce the inefficiencies of TD respondents while increasing the efficiency of DP respondents. Off-farm income not only decreases the availability of labor but also the incomes are not used sufficiently for on-farm activities of TD while DP respondents use their off-farm income in the field (Abdulai and Huffman, 2000; Kramol et al., 2013). Similarly, the tenancy status of the growers also increases the efficiency level in the study area. Chen (2017) stated that ownership (titled land) can encourage agricultural production. Television contributes a significant role in the efficiency of respondents. Farmer's access to television can get information related to agriculture, technology, climate, and others, and their best practices increase their efficiency (Areal et al., 2020). In the current study, agricultural credit and livestock have no impact on the efficiency level of farmers.

### GHG Emissions From Urea Manufacture for Maize

Nitrogen is one of the key factors for maize growth, yield, and profitability (Khan et al., 2008). By considering the application rate (Urea ^*^ 0.46 = nitrogen) of nitrogen by individuals, the GHG emission from the manufacturing of synthetic N consumed by top-dressing and deep placement fertilization are shown in Table 10. The results show that the total CO_2_ emitted from the manufacturing of synthetic N used by TD farmers was 29.716 MtCO_2_ equal while the CO_2_ emitted from the manufacturing of N used by DP farmers was 21.443 MtCO_2−_. The estimated GHG emission shows that 8.273 MtCO_2_ of carbon can be reduced from the manufacturing of synthetic N if the farmers of TD shift toward deep placement fertilization. Similarly, the average GHG emission by the TD and DP farmers was 237.72 and 171.54 kgCO_2_/ha, respectively, which implies that each farmer of the DP group can reduce GHG emission by 66.18 kgCO_2_/ha from the manufacturing of synthetic N if they shift toward deep placement. The estimated results are similar to the previous study by Chai et al. (2019). The estimated results of OLS show that area and urea have a significant impact on the manufacturing emission at a 1% significant level. An increase of 1 unit in area and urea can increase the manufacturing emission by 108.70 and 1.50 kgCO_2_.

**Table 10:** GHG emissions from the manufacturing of urea and OLS.

	**Top Dressing**	**Deep placement**	**Difference**
Total (MtCO_2_)	29.716	21.443	8.273
Average (kgCO_2_)	237.72	171.54	66.18
Standard deviation	158.36	117.58	
Minimum (kgCO_2_)	32.224	19.458	
Maximum (kgCO_2_)	687.24	513.91	
**Ordinary Least Square for the manufacturing of Urea**			
	Coeff (Std. Err)	Coeff (Std. Err)	
Constant	−228.53* (11.82)	−170.66*(7.65)	
Area (hectare)	108.70* (3.70)	77.69*(2.63)	
Urea (kg)	1.50*(0.06)	1.60*(0.06)	

*Survey data, (2021)*.

### Direct GHG Emissions From Urea Fertilization

The use of synthetic N in the cropland emits GHG in the form of N_2_O–N. To convert the N to N_2_O, the estimated results are multiplied with the fraction 44/28. The results shown in able 11 display that the total fertilization of synthetic N applied to the maize crops by TD and DP respondents was 311.31 and 224.64 kgN_2_O. The projected result implies that TD respondents can reduce 86.67 kgN_2_O by shifting toward deep placement fertilization. Similarly, the average GHG emission due to the fertilization of synthetic N emits 2.49 and 1.80 kgN_2_O/ha. The difference between the two methods of fertilization shows that if the farmers used deep placement fertilization of synthetic N instead of top-dressing fertilization, the GHG emission can reduce by 0.12 kgN_2_O/ha. The estimated results are similar to the previous studies of Liu et al. (2006) and Signor et al. (2013), which stated that deep placement fertilization is the finest technique of mitigation of N_2_O soil emission. Correspondingly, Gaihre et al. (2016) stated that N_2_O emission from deep placement is 80% less than top dressing. The estimated results of OLS show that area and synthetic N have a significant impact on the manufacturing emission at a 1% significant level. An increase of 1 unit in area and synthetic N can increase the manufacturing emission by 1.14 and 0.03 kg of N_2_O.

Similarly, the study also measures the yield base GHG emission from the manufacturing and direct use of synthetic N in the respondent area. The projected results in Table 11 show that the area under TD and DP respondents emit a total of 37.81 and 21.95 kgCO_2_ from the total grain produced. From the estimated results it is clear that 15.86 kgCO_2_ can be reduced from the total yield produced by TD respondents. Likewise, the average GHG emissions from the manufacturing and direct use of N can be reduced by 0.12 kgCO_2_ from 1 kg of maize grain. The average GHG emission from the TD and DP maize respondents was 0.30 and 0.18 kgCO_2_/kg of grain. Likewise, the area-based GHG emission from the manufacturing and direct use of N shows that the total GHG emission from the area used by TD and DP respondents was 53.41 and 38.69 MtCO_2_. The projected results show that TD respondents emit 14.72 MtCO_2_ more than DP respondents. The average GHG emission from a single hectare of land for TD and DP was 427.30 and 309.51 kgCO_2_/ha. The result implies that TD respondents can reduce GHG emissions up to 117.79 kgCO_2_/ha by shifting from top-dressing fertilization to deep placement fertilization.

**Table 11:** Direct, OLS, yield, and area-scaled GHG emissions from synthetic N.

**Direct GHG emissions from synthetic N**
	**Top dressing**	**Deep placement**	**Difference**
Total (kgN_2_O)	311.31	224.64	86.67
Average (kg N_2_O/ha)	2.49	1.80	0.69
Standard deviation (kg N_2_O/ha)	1.66	1.23	
Minimum (kg N_2_O/ha)	0.36	0.20	
Maximum (kg N_2_O/ha)	7.20	5.38	
**Ordinary Least Square for the manufacturing of Urea**			
	Coeff (Std. Err)	Coeff (Std. Err)	
Constant	−2.39* (0.12)	−1.79*(0.08)	
Area (hectare)	1.14* (0.04)	0.81*(0.03)	
Synthetic N (Kg)	0.03* (0.001)	0.04*(0.001)	
**Yield-scaled GHG emissions from manufacturing and direct use of synthetic N**			
Total (KgCO_2_)	37.81	21.95	15.86
Average (KgCO_2_/Kg of grain)	0.30	0.18	0.12
Standard deviation (KgCO_2_/kg of grain)	0.18	0.11	
Minimum (KgCO_2_/kg of grain)	0.06	0.03	
Maximum (KgCO_2_/kg of grain)	0.77	0.46	
**Area-scaled GHG emissions from manufacturing and direct use of synthetic N**			
Total (MtCO_2_)	53.41	38.69	14.72
Average (KgCO_2_/Ha)	427.30	309.51	117.79
Standard deviation (KgCO_2_/Ha)	167.06	112.55	
Minimum (KgCO_2_/Ha)	176.34	133.67	
Maximum(KgCO_2_/Ha)	708.18	568.82	

*Survey data (2021)*.

## Discussion

Farm profitability and productivity are the most important aim of farmers. Therefore, this study was carried out to differentiate the TD and DP fertilization methods based on their profitability, nitrogen efficiency, and greenhouse emission from the use of synthetic nitrogen (Urea). The study was based on primary data collected from different respondents and areas through a well-designed questionnaire. It is observed that the net revenue obtained from the top dressing was 71,187.8 PKR/ha while net revenue gained by side-dressing farmers was 172,087.2 PKR/ha. The profitability ratio portrays that the deep placement fertilization method was a more profitable and least cost method as compared to the top-dressing fertilization. Similarly, by using the SFA model, the study also observed that the seed rate used by TD and DP groups are using quality seed of maize but the elasticity of seed for TD is more than DP, which implies that the seed quality and seedbed of TD is better than DP (Saddozai et al., 2015; Nasrullah et al., 2021a,b). Likewise, the number of labor used for TD and DP also shows a significant improvement in maize production. The significant impact of labor shows the better management, skill, and knowledge of labor used by the farmers (Ali et al., 2019; Jo et al., 2021).

The area used by the respondents in the study area shows that if the farmers increase their farmland can significantly improve productivity and efficiency. The farmer with more land pays more attention to farming. Zulfiqar et al. (2020a) and Nasrullah et al. (2019) stated that soil fertility and better management of land can increase production. Likewise, the irrigation application in the projected area is also significant for both TD and DP. The on-time irrigation can increase productivity while water stress can decrease the maize production and kernel size (Traore et al., 2000; Payero et al., 2009). The Farmyard manure also shows a substantial rise in production by providing micronutrients (Naveed et al., 2018). The DAP used by the respondents has significantly improved the maize yield (Chen et al., 2020). Similarly, the impact of urea is also statistically significant at a 1% significance level. The result indicates that an accurate amount of fertilizer was used at a suitable subsidized price (Ali et al., 2019). The tractor used for seedbed preparation has an effective impact on the production of maize in the study area. The machinery used for inverting and plowing land had a direct relation with production (Sekhon et al., 2010; Nasrullah et al., 2019). Likewise, pesticides used by respondents in the study area significantly improve maize production because pests and insects can reduce production (Popp et al., 2013).

The demographic, institutional, and socioeconomic factors are the important factors, which cannot be ignored when measuring the efficiency level of farmers. The inefficiency factor age has a significant impact on the efficiency level of maize growers in the TD or DP groups. The increase in age showed the weak physical condition of the growers (Saddozai et al., 2015). Similarly, the education level of maize growers shows that the improved skills of the farmers, which is a key indicator of achieving improved technology and getting maximum efficiency (Dhungana et al., 2004; Mariano et al., 2010) while Rios and Shively (2005) stated that higher education decreases the efficiency level.

Nitrogen is one of the key factors for maize growth, yield, and profitability (Khan et al., 2008). By considering the application rate of nitrogen by individuals, the GHG emission from the manufacturing of synthetic N consumed by top-dressing and deep placement fertilization was 29.716 and 21.443 MtCO_2−_. It is observed that carbon emissions of TD fertilization were more than that of DP fertilization. Similarly, the estimated results of OLS show that an increase of 1 unit in area and urea can increase the manufacturing emission by 108.70 and 1.50 kgCO_2_. The use of synthetic N in the cropland emits GHG in the form of N_2_O–N. To convert the N to N_2_O the estimated results are multiplied with the fraction 44/28. The projected result implies that TD respondents produce more GHG emissions than deep placement fertilization. Liu et al. (2006) and Signor et al. (2013) stated that deep placement fertilization is the finest technique of mitigation of N_2_O soil emission.

## Conclusions

The study was based on primary data in which the data are collected from different respondents and different areas using the TD and DP fertilization method through a well-designed questionnaire. It is concluded from the study that the TD method is costly as compared to the DP fertilization technique. It is observed that the fertilizer used by TD is more as compared to the DP group. It implies that during TD fertilization, most of the fertilizers fall on the leaves of maize plants which causes the burning of leaves, and reduces the yield and efficiency of nitrogen used. Similarly, the profitability ratio shows that both TD and DP respondents are using almost the same amount of inputs except urea. The cost per hectare incurred by the TD groups is more than the DP groups. Similarly, the yield of the TD growers is 727.82 kg/ha more than that of TD respondents. Therefore, if the farmers of TD move toward DP, they can increase their profit up to 34.47 thousand rupees per hectare. The maximum likelihood estimation shows that all the variable inputs used by both groups significantly improved maize production. The efficiency score shows that the average efficiency level of TD and DP respondents are 87% and 90%. To reach the 90% efficiency level, the farmers of TD need to use deep placement fertilization. Correspondingly, the gap between the mean efficiency and maximum efficiency score of the TD and DP groups also shows that the farmers of TD and DP can increase their efficiency up to 12% and 9% by using the same inputs. The findings also concluded that the GHG emission from the manufacturing of synthetic N during TD fertilization is more than that of DP. Similarly, it is also clear that Yield and Area-Scaled GHG emissions from the direct use of synthetic N of TD are greater than that of the DP group. During the top-dressing fertilization, most of the fertilizer fall on surface land unwantedly which easily diffuses to N_2_O–N as compared to the DP fertilization. This unwanted diffusion of synthetic N is not only harmful to the environment but also can increase the cost and decreases the yield of respondents.

Based on the study findings, it is suggested that

1) DP fertilization has a less harmful effect than TD fertilization on the environment. Therefore, special training and programs are needed for the farmers' community to reduce GHG emissions.
2) All the inputs used by the respondents showed a significant impact on maize production. Therefore, for increasing the farmer's yield, profit, efficiency, and food security, farmers need to use all the inputs in a better way to maximize their production.
3) Based on the efficiency score in the respondent area, the farmers need to adopt a new technique, seed quality, and seedbed to get maximum efficiency with the given level of inputs.
4) Based on the profitability ratio, farmers' efficiency level, and GHG emissions, it is suggested that farmers need to prefer DP fertilization instead of TD to get maximum profit and yield with low environmental damage.

## Data Availability Statement

The raw data supporting the conclusions of this article will be made available by the authors, without undue reservation.

## Author Contributions

MN: conceptualization, formal analysis, investigation, and writing the original draft. MR: data collection and software. XY: methodology and formal analysis. LL: supervision, investigation, and data correction. AM, HA, BA, and SF: formal analysis, methodology, and editing. All authors contributed to the article and approved the submitted version.

## Funding

This research was sponsored by the Innovation Platform Open Fund Project of Hunan Education Department, China (19K087) and Researchers Supporting Project Number (TURSP2020/110), Taif University, Taif, Saudi Arabia.

## Conflict of Interest

The authors declare that the research was conducted in the absence of any commercial or financial relationships that could be construed as a potential conflict of interest.

## Publisher's Note

All claims expressed in this article are solely those of the authors and do not necessarily represent those of their affiliated organizations, or those of the publisher, the editors and the reviewers. Any product that may be evaluated in this article, or claim that may be made by its manufacturer, is not guaranteed or endorsed by the publisher.
